# Polyprotein-Driven Formation of Two Interdependent Sets of Complexes Supporting Hepatitis C Virus Genome Replication

**DOI:** 10.1128/JVI.01931-15

**Published:** 2016-02-26

**Authors:** Rafael G. B. Gomes, Olaf Isken, Norbert Tautz, John McLauchlan, Christopher J. McCormick

**Affiliations:** a Division of Clinical and Experimental Sciences and Institute for Life Sciences, Faculty of Medicine, University of Southampton, Southampton, United Kingdom; b Institute of Virology and Cell Biology, University of Lübeck, Lübeck, Germany; c MRC-University of Glasgow Centre for Virus Research, Glasgow, United Kingdom

## Abstract

Hepatitis C virus (HCV) requires proteins from the NS3-NS5B polyprotein to create a replicase unit for replication of its genome. The replicase proteins form membranous compartments in cells to facilitate replication, but little is known about their functional organization within these structures. We recently reported on intragenomic replicons, bicistronic viral transcripts expressing an authentic replicase from open reading frame 2 (ORF2) and a second duplicate nonstructural (NS) polyprotein from ORF1. Using these constructs and other methods, we have assessed the polyprotein requirements for rescue of different lethal point mutations across NS3-5B. Mutations readily tractable to rescue broadly fell into two groupings: those requiring expression of a minimum NS3-5A and those requiring expression of a minimum NS3-5B polyprotein. A *cis*-acting mutation that blocked NS3 helicase activity, T1299A, was tolerated when introduced into either ORF within the intragenomic replicon, but unlike many other mutations required the other ORF to express a functional NS3-5B. Three mutations were identified as more refractile to rescue: one that blocked cleavage of the NS4B5A boundary (S1977P), another in the NS3 helicase (K1240N), and a third in NS4A (V1665G). Introduced into ORF1, these exhibited a dominant negative phenotype, but with K1240N inhibiting replication as a minimum NS3-5A polyprotein whereas V1665G and S1977P only impaired replication as a NS3-5B polyprotein. Furthermore, an S1977P-mutated NS3-5A polyprotein complemented other defects shown to be dependent on NS3-5A for rescue. Overall, our findings suggest the existence of two interdependent sets of protein complexes supporting RNA replication, distinguishable by the minimum polyprotein requirement needed for their formation.

**IMPORTANCE** Positive-strand RNA viruses reshape the intracellular membranes of cells to form a compartment within which to replicate their genome, but little is known about the functional organization of viral proteins within this structure. We have complemented protein-encoded defects in HCV by constructing subgenomic HCV transcripts capable of simultaneously expressing both a mutated and functional polyprotein precursor needed for RNA genome replication (intragenomic replicons). Our results reveal that HCV relies on two interdependent sets of protein complexes to support viral replication. They also show that the intragenomic replicon offers a unique way to study replication complex assembly, as it enables improved composite polyprotein complex formation compared to traditional *trans*-complementation systems. Finally, the differential behavior of distinct NS3 helicase knockout mutations hints that certain conformations of this enzyme might be particularly deleterious for replication.

## INTRODUCTION

Exposure to hepatitis C virus (HCV) often results in chronic infection and can lead to liver-related mortality and morbidity. Intensive research efforts since the discovery of the virus have translated into improved patient care, with the recent advent of direct acting antiviral (DAA) therapy now providing high cure rates, particularly in genotype 1 (gt1)-infected patients (1). However, certain aspects of viral replication remain poorly understood, including how viral proteins associate with each other and host proteins to form the platforms that support replication of the viral genome.

HCV has a single-stranded, positive-sense RNA genome of 9.6 kb in length that contains a single open reading frame (ORF) flanked by 5′ and 3′ untranslated regions (UTRs) (2). Translation of the ORF is driven by an internal ribosome entry site (IRES) within the 5′ UTR (3), resulting in the production of a polyprotein that is cleaved co- and posttranslationally by host- and virus-encoded proteases to give rise to the mature viral proteins. The latter two-thirds of the polyprotein encompass the NS3-5B replicase unit (NS3, NS4A, NS4B, NS5A, and NS5B), which provides the viral proteins that are sufficient for genome replication (4). NS3 is the protease responsible for NS3-5B processing and also provides essential helicase activity, encoded within its C-terminal domain (5). It is anchored to the endoplasmic reticulum (ER) membrane both by an amphipathic helix and by its protease cofactor NS4A (5). RNA polymerase activity is provided by NS5B (6). The functions of NS4B and NS5A are less well understood, although both proteins bind RNA (7, 8).

In infected cells, viral RNA synthesis occurs in virus-induced cytoplasmic compartments referred to as membranous webs or membrane-associated foci (MAF) (9). Morphologically, these compartments appear as a collection of ER-derived single- and double-membrane vesicles (10). Current evidence points to the double-membrane vesicles as the site for polymerase activity and hence RNA replication (11). Expression of polyproteins encoding NS3-5A or NS3-5B leads to the formation of MAF-like structures in the absence of replication (10). Double-membrane vesicles are also present in cells expressing NS5A alone (10), and both drugs and mutations that target NS4B and NS5A disrupt MAF architecture (12–15); this suggests that NS4B and NS5A are key structural components of these assemblies.

Genetic complementation of mutations that block replication offers a way to gain insight into the functions of viral NS proteins in RNA replication. From early studies with gt1b replicons, most mutations in the HCV replicase were refractile to rescue, except for a few mutations in the low-complexity sequence 1 (LCS1) region between domains 1 and 2 in NS5A (16). Subsequent analysis using replicons encoding strain JFH-1 (gt2a) sequences, which give higher levels of replication, revealed that mutations in other NS5A regions as well as NS4B mutations could be rescued, albeit less efficiently than LCS1 mutations (14, 17). The range of mutations that can be rescued has recently been extended to include NS3, NS4A, and NS5B by using Venezuelan equine encephalitis virus (VEEV) replicons to express high levels of NS3-5B (18).

All of the above approaches have relied on supplying functional counterparts of proteins in *trans* that are expressed from separate transcripts to rescue defective NS3-5B replicase units. Recently, we reported an alternative system to complement deleterious mutations using intragenomic HCV replicons that carry two ORFs (ORF1 and ORF2), each driven by separate IRES elements. Thus, the complementing viral proteins are provided in *trans* from distinct translation units but encoded in *cis* from the same RNA molecule. Two advantages of our system were that (i) complementing proteins were translated to a level similar to that of their nonfunctional counterpart and (ii) functional and nonfunctional polyproteins could be translated in close apposition. Using this approach, enhanced rescue of defective NS5A containing an S2208I mutation could be achieved by expressing an NS3-5A cassette in ORF1 compared to expressing NS5A alone (19). Enhanced rescue of this defective NS5A also correlated with the ability of NS3-5A to target the protein to MAF. However, despite the efficiency with which NS3-5A targeted NS proteins to the MAF, both its ability and the capacity of helper viruses expressing NS3-5B to complement NS4B defects remained limited.

In this study, we set out to understand why rescue of certain mutations remained limited, despite efficient targeting of NS proteins to the MAF by NS3-5A using the intragenomic replicon system (19). By extending the capacity of ORF1 to express NS3-5B, our data reveal the existence of genetically distinguishable complexes that support HCV RNA replication.

## MATERIALS AND METHODS

### Cell lines.

Generation of the SGR-JFH1neo replicon cell line (where SGR is subgenomic replicon) has been described, as has propagation of this cell line and naive Huh7.5 cells (19, 20).

### Plasmid vectors.

The production of pFBM-based baculovirus constructs for expression of JFH1 NS34A, green fluorescent protein (GFP)-tagged versions of JFH1 NS5A expressed either alone or in the context of NS3-5A and NS3-5B, pSGR-FLAG constructs containing G1911A and S2208I mutations, and intragenomic replicon-containing plasmids pRep_R2NS5A^V5^/NS3-5B^FLAG^ and pRep_R2NS3-5A^V5^/NS3-5B^FLAG^ with either a functional ORF2 or an ORF2 containing S2208I and G1911A have been described (19).

To extend intragenomic replicon ORF1 to express a Renilla-foot-and-mouth disease virus 2A (FMDV2A)-NS3-5B polyprotein containing a V5 epitope tag at the COOH terminus of NS5A, two 1,000-bp DNA strings (Invitrogen) representing an entire synonymous codon-altered NS5B sequence + NS5A5B boundary and flanked at the 3′ end by a stop codon, PmeI site and M13(−20) primer site were synthesized. These DNA strings along with LIT_Ren-2A-based vector containing the codon-altered NS3-5A^V5^ sequence (LIT_Ren-2A-NS3-5A^V5^) (19) were used as the templates in 3 separate PCRs involving primer pairs 1 + 2, 3 + 4, and 5 + 6 (see Table S1 in the supplemental material). All three products were combined in a second PCR with primers 1 and 6, and the resulting DNA was cloned back into LIT_Ren-2A-NS3-5A^V5^ via SalI and PmeI restriction sites, forming LIT_Ren-2A-NS3-5B^V5^ [for the codon-altered NS3-5B(5A^V5^) sequence, see GenBank accession number KR140016]. The Ren-2A-NS3-5B^V5^ expression cassette from this vector was cloned into existing pRep_R2NS3-5A^V5^/NS3-5B^FLAG^-based vectors via BglII and PmeI restriction sites to generate pRep_R2NS3-5B^V5^/NS3-5B^FLAG^ constructs, where ORF2 either was functional or contained the ΔGDD, G1911A, or S2208I mutation. The terminology in the main body of text used to describe the transcripts generated from these constructs is wt^NS3-5B^/wt^NS3-5B^, wt^NS3-5B^/ΔGDD^NS3-5B^, etc. (where wt is wild type).

To produce intragenomic replicons expressing Ren-2A-NS34A, Ren-2A-NS5A5B, and Ren-2A-NS5B cassettes, LIT_Ren-2A-NS3-5B^V5^ was used as a template in a PCR with primers 7 + 8, 9 + 6, and 10 + 6, respectively. The resulting NS coding regions were transferred to a LIT_Ren-2A-based vector using RsrII and PmeI restriction sites to produce LIT_Ren-2A-NS34A, LIT_Ren-2A_NS5A5B, and LIT_Ren-2A-NS5B. The expression cassettes from these vectors were cloned into existing intragenomic replicon-containing plasmids via BglII and PmeI restriction sites.

Introduction of the L1157A mutation into pSGR-FLAG (19) was through exchange of an Acc65I-SpeI restriction fragment with that contained within the pCITE-NS3-3′/JFH1 vector containing this same mutation (2). Introduction of the S1977P mutation into pSGR-FLAG was through exchange of an NsiI-RsrII restriction fragment with that present in pFBM(NS3-5A^GFP^)S1977P. Introduction of the other lethal mutations into pSGR-FLAG relied on a two-step PCR using either JFH1 cDNA or pSGR-FLAG as the template. Appropriate flanking primers were used in combination with internal mutagenic primers 11 + 12 (for PP1220-1GG), 13 + 14 (for K1240N), 15 + 16 (for V1665G), 17 + 18 (for Y1706A), 19 + 20 (for S1977P), 21 + 22 (for P2008A), 23 + 24 (for GDD>GAA), 25 + 26 (T1299A), and 27 + 28 (G2313A). [Note that for the G2323A mutation, a JFH1 template with a small number of silent substitutions was used. This contained a change to the BamHI site within NS5A to GGGTCC and substitutions in a 10-nucleotide poly(C) tract adjacent to the mutagenized codon to allow an effective overlapping primer design. Fragments generated were cloned into pSGR-FLAG via ClaI and SpeI (K1240N), ClaI and NsiI (PP1220-1GG + T1299A), NsiI and RsrII (V1665G + Y1706A + P2008A + S1977P), SfiI and SnaBI (GDD>GAA), or BamH I and RsrII (G2313A). Transfer of mutations from pSGR-FLAG into ORF2 of intragenomic replicon-containing plasmids took advantage of a set of unique restriction sites in a region of sequence identity shared by the two constructs, between the start of the encephalomyocarditis virus (EMCV) IRES through to the end of the 3′ UTR.

Mutations in ORF1 of plasmids containing the intragenomic replicons were introduced by a two-step PCR approach using LIT_Ren-2A-NS3-5A^V5^ and/or LIT_Ren-2A-NS3-5B^V5^ as the templates. Appropriate flanking primers were used in combination with internal mutagenic primers 29 + 30 (for K1240N), 31 + 32 (for S1977P), 33 + 34 (for GDD>GAA), 35 + 36 (for T1299A), 37 + 38 (for G1911A), and 39 + 40 (for V1665G). The resultant DNA was cloned back into LIT_Ren-2A-NS3-5A^V5^ and/or LIT_Ren-2A-NS3-5B^V5^ via either RsrII and MluI (K1240N), NdeI and SalI (G1911A + S1977P), SalI and PmeI (GDD>GAA), or NotI + MluI (T1299A) restriction sites. The same approach was used to introduce the K1240N mutation into LIT_Ren-2A-NS34A, but with the PCR product being cloned back into this plasmid via RsrII and PmeI sites. The expression cassettes from these vectors were cloned into existing intragenomic replicon-containing plasmids via BglII and PmeI restriction sites. In the case of the V1665G mutation, cloning was directly into the intragenomic vector via NotI + MluI.

To generate monocistronic replicons, pSGR-FLAG was used as a template in a PCR with primers 41 and 42, and the resulting product was cloned into pRep_R2NS3-5A^V5^/NS3-5B^FLAG^ via the two RsrII sites present within the vector to generate pJFH1-mono (the DNA template for transcription of JFH1-mono). Both pRep_R2NS3-5B^V5^/NS3-5B^FLAG^ and pJFH1-mono were used as the templates in a two-step PCR with primer pairs 43 + 44 and 45 + 46. The resultant DNA, produced in the second-round reaction with primers 43 and 46, was cloned into pJFH1-mono via AscI and SalI sites to generate the pDVR/JFH1@7667-mono.

### Sequence analysis.

Analysis of the synonymous codon-altered sequence to establish the CpG and UpA motif frequencies was performed using Simple Sequence Editor software (21).

### Transient replication assays.

Generation of T7 RNA transcripts and their electroporation into Huh7.5 cells or into Huh7.5 cells previously transduced with baculovirus has been described (19). For all assays except those looking at the *trans*-dominant impact of K1240N on replication, cells were electroporated with 2 μg of RNA transcript. In the case of the latter set of assays, 1 μg of replication-competent JFH1-mono was transfected with 4 μg of either yeast tRNA or the replication-defective bicistronic SGR replicon. Cell lysates were harvested in 1× passive lysis buffer (Promega) up to 96 h posttransfection, and luciferase activity was measured. Firefly and Renilla luciferase activities were measured using the Bright-Glo Luciferase Assay system (Promega) and Renilla Luciferase Assay kit (Biotium) according to the manufacturers' recommendations. For graphical representation, the background signal from mixing passive lysis buffer only with luciferase reagent was subtracted from the raw data values. On some occasions where different experiments containing multiple experimental groups were performed in parallel, control experimental groups were shared between experiments (indicated in figure legends).

### Western blot analysis.

Transfer of proteins to membranes and subsequent detection have been described (19). The antibodies used were anti-NS3 (BioFront), anti-glyceraldehyde-3-phosphate dehydrogenase (anti-GAPDH; Chemicon), anti-V5 (gift from R. Randall, University of St. Andrews), anti-FLAG (Biolegend), and anti-GFP (Biolegend).

### Indirect immunofluorescence.

Seventy-two hours posttransfection, cells were fixed with methanol at −20°C for 30 min, rehydrated with phosphate-buffered saline (PBS), and blocked with PBS containing 2% fetal calf serum (PBS/FCS) for 10 min. Cells were then probed with anti-V5 and anti-FLAG antibodies for 1.5 h at room temperature, washed extensively with PBS/FCS, and finally incubated with anti-mouse-Alexa Fluor 594 and anti-rat-Alexa Fluor 488 for 1 h at room temperature. All antibody incubations were carried out in PBS/FCS. Cells were washed with PBS/FCS followed by PBS, rinsed with H_2_O, and then mounted with Vectashield (Vector Laboratories) that contained DAPI (4′,6-diamidino-2-phenylindole) to stain nuclei. Images were captured with a Zeiss LSM 710 confocal microscope.

### Northern blot analysis.

Cellular RNA, extracted using TriFAST reagent (Peqlabs), was run on a formaldehyde-agarose gel and transferred by capillary action to a positively charged nylon membrane using standard procedures. DNA probes were biotinylated using the PlatinumBright labeling kit (Kreatech). Hybridization was performed using Ultrahyb (Ambion) according to the manufacturer's instructions, and bound probe was detected using the Bright Star detection kit (Ambion) and film-based exposure.

## RESULTS

### Replication of HCV RNA from two copies of the NS3-5B replicase unit in a single RNA molecule.

**FIG 1:**
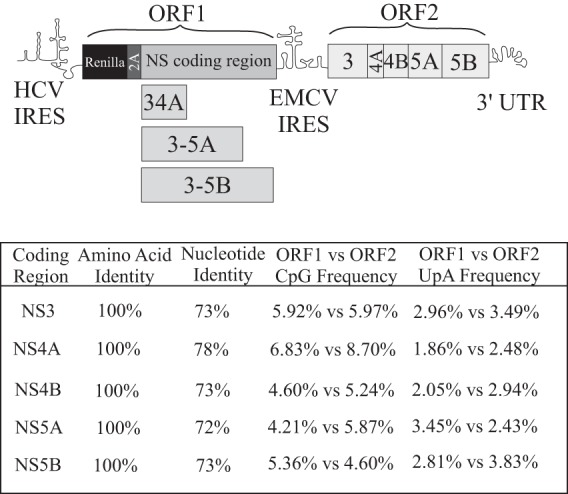
Schematic representation of the intragenomic HCV replicons used in this study. The HCV IRES in the 5′ UTR drives translation of ORF1, which encodes the indicated NS polyproteins expressed as part of a Renilla-foot and mouth disease (FMDV) 2A fusion protein. An encephalomyocarditis virus (EMCV) IRES drives translation of the NS3-5B replicase in ORF2. When present, NS5A is expressed from ORF1 as a COOH-terminal V5 epitope-tagged protein. Constructs express NS5A from ORF2 as a FLAG-tagged protein. Coding regions in ORF1 downstream from Renilla 2A represent recoded NS sequences incorporating synonymous sequence alterations compared to the authentic viral counterpart sequence present in ORF2. The table compares the amino acid and nucleotide sequences as well as the CpG and UpA frequencies in ORF1 (synthetic sequence) and ORF2 (authentic viral sequences).

**FIG 2:**
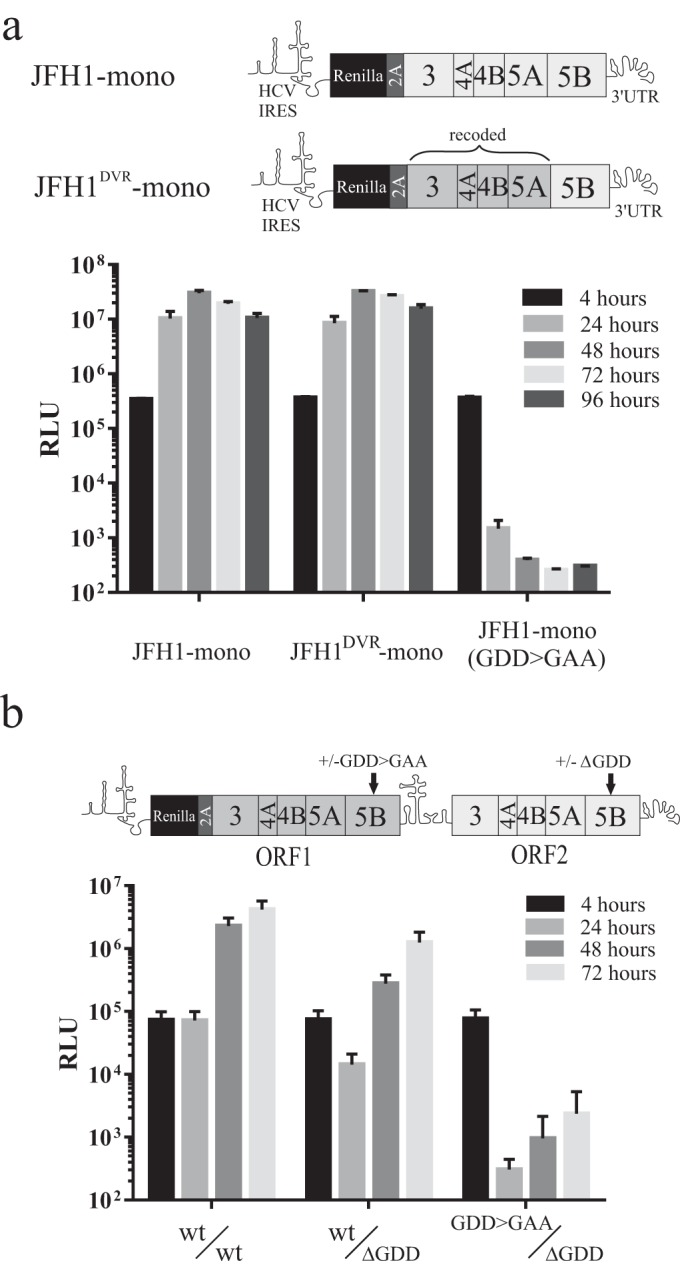
Replication of constructs expressing NS3-5B as a Renilla-FMDV 2A fusion protein. Replication was assessed using both monocistronic (a) and intragenomic (b) replicon constructs. The schematic above each graph indicates the regions of NS3-5B encoded by the recoded and authentic JFH1 coding sequences for each construct. Data represent the means ± standard deviations (SD) from two independent experiments (a) and means ± standard errors of the means (SEM) from three independent experiments (b).

We recently described the use of intragenomic replicons as a novel system to perform HCV complementation assays (Fig. 1) (19). Using these constructs, we showed that the intracellular distribution of a V5-tagged NS5A, expressed from ORF1 as an NS3-5A polyprotein, extensively overlapped a second copy of a FLAG-tagged NS5A produced from an ORF2-encoded replicase. Along with additional experimental data, the results were consistent with NS3-5A directing mature protein products from the polyprotein to MAF but did not account for why expression of NS3-5A from ORF1 allowed rescue of constructs carrying only the NS5A S2208I mutation in ORF2 and not an NS4B G1911A mutation (the numbers refer to the position of the residue in the JFH1 polyprotein). One possible explanation was the existence of more than one type of NS protein complex within MAF such that NS3-5A, being an incomplete component of the full NS3-5B replicase, contributed to the formation of only some of these complexes. Two alternative explanations were that linking the ORF1 polyprotein to a Renilla luciferase-foot-and-mouth disease virus (FMDV) 2A fusion protein or the use of a recoded NS sequence in ORF1 impacted on polyprotein function. To examine these two possibilities, monocistronic replicons in which the NS3-5B replicase was expressed as a Renilla-FMDV2A fusion protein were generated (Fig. 2a). For one replicon, the NS protein coding region was derived from JFH1 (JFH1-mono), but for the other it was chimeric, consisting of the NS3-5A recoded region fused to JFH1 NS5B (JFH1^DVR^-mono). Expressed polyproteins also encoded the same FLAG-tagged (JFH1-mono) and V5-tagged (JFH1^DVR^-mono) versions of NS5A present in ORF2 and ORF1 of the intragenomic replicon construct, the former already having been shown not to affect replicative function (19). The two replicons showed equally robust replication when transfected into cells, whereas a polymerase-defective control monocistronic construct containing a GDD>GAA mutation [JFH1-mono(GDD>GAA)] did not replicate (Fig. 2a). These data verify that neither the linking of a luciferase reporter to NS3-5B using FMDV 2A nor the use of recoded NS3-5A sequence interferes with RNA replication. They also demonstrate that the presence of the V5 tag in recoded NS5A does not impair replicase function.

To determine whether intragenomic replicons could be adapted for identifying distinct replication complexes (RCs), we then extended NS3-5A in ORF1 to encode NS3-5B such that the synthetic NS5B sequence, similar to the upstream NS3-5A segment, also contained synonymous changes across its coding region. The strategy to introduce synonymous changes was aimed at limiting the possibility of recombination with NS3-5B in ORF2 and had the added advantage of preventing duplication of *cis*-acting RNA elements (CREs) present in ORF2 (22, 23). Transfection of the resultant wt^NS3-5B^/wt^NS3-5B^ RNA into Huh7.5 cells gave robust replication (Fig. 2b). To test whether the codon-modified NS3-5B sequences in ORF1 could direct replication, the GDD motif in the NS5B RNA polymerase in ORF2 was deleted (ΔGDD). The resultant intragenomic construct (wt^NS3-5B^/ΔGDD^NS3-5B^) gave luciferase activity at 72 h that was only about 3-fold lower than that of the replicon with wt sequences in ORFs 1 and 2. Combining another polymerase knockout mutation (GDD>GAA) in ORF1 with the ΔGDD mutation in ORF2 (giving GDD>GAA^NS3-5B^/ΔGDD^NS3-5B^) blocked replication (Fig. 2b). These data demonstrate that it is possible to express two functional copies of the HCV NS3-5B replicase unit from a single RNA molecule.

### Mutations in NS3, NS4B, and NS5A that are refractile to rescue with NS3-5A.

**FIG 3:**
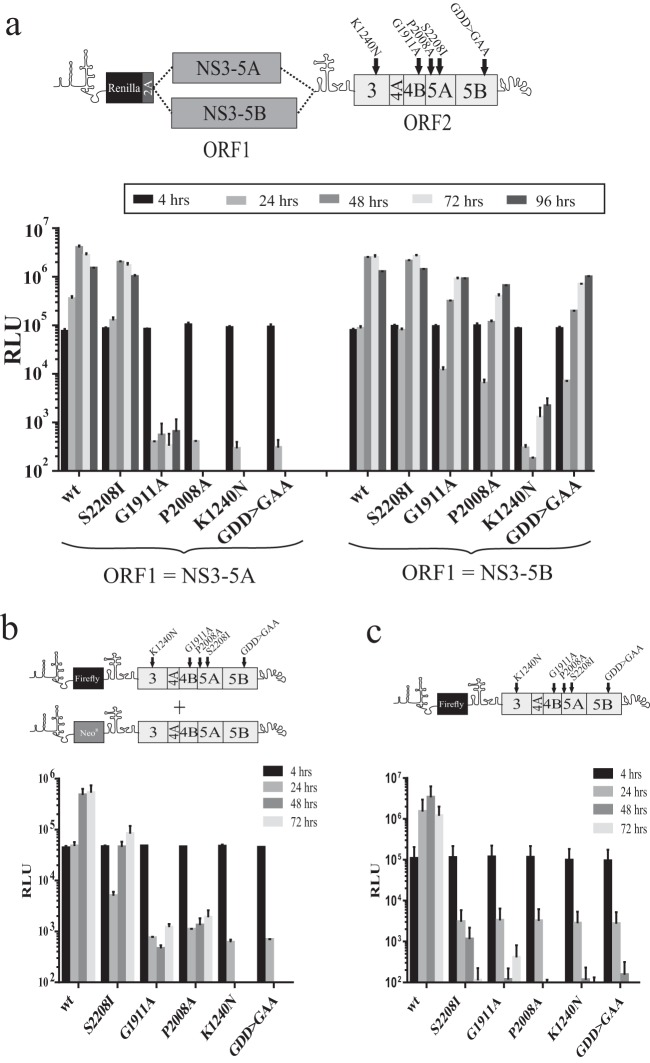
Assessing the capacity of ORF1 polyproteins to support replication of intragenomic replicons with different defects in ORF2 and recue of the same defects by *trans*-complementation. (a) Replication assay data of intragenomic replicons expressing functional NS3-5A or NS3-5B in ORF1 and expressing NS3-5B carrying a lethal mutation in ORF2 as defined in the schematic above the graph. (b and c) These mutations were introduced into bicistronic replicons expressing firefly luciferase in ORF1, and the capacity of these constructs to replicate was assessed in a stable neomycin-resistant (Neo^r^) helper replicon cell line (b) or in naive Huh7.5 cells (c). Data represent the means ± SD from two independent experiments.

To examine the effect of introducing other lethal mutations into ORF2 of the intragenomic replicon, constructs expressing either wt NS3-5A or NS3-5B from ORF1 and a series of NS3-5B replicases carrying single lethal mutations from ORF2 were made (Fig. 3). The first two of these mutations were the S2208I NS5A and G1911A NS4B mutations from our previous study (19), in which only S2208I could be complemented by NS3-5A expressed from ORF1. The third was another mutation in NS5A (P2008A), previously reported to be rescued by *trans*-complementation and chosen because it belonged to a different complementation group from the S2208I mutation (17). The final mutation was K1240N, located in the Walker A motif of the NS3 helicase and which blocks helicase activity by preventing nucleoside triphosphate (NTP) binding.

This series of replicons and relevant polymerase-defective control constructs (wt^NS3-5A^/GDD>GAA^NS3-5B^) were transfected into Huh7.5 cells, and luciferase activity was monitored over a 96-h period (Fig. 3). Replication was slightly lower for wt^NS3-5B^/wt^NS3-5B^ than for wt^NS3-5A^/wt^NS3-5B^, which may have been due to the increased length of the RNA from incorporating NS5B sequences into ORF1 of wt^NS3-5A^/wt^NS3-5B^. As predicted, wt^NS3-5A^/GDD>GAA^NS3-5B^ did not replicate, as the RNA expresses only a nonfunctional virus-encoded RNA polymerase. Similar to the results from wt^NS3-5B^/ΔGDD^NS3-5B^ shown in Fig. 2, wt^NS3-5B^/GDD>GAA^NS3-5B^ also showed robust replication. Results with the constructs containing other mutations in ORF2 revealed differences in replication between the experimental groups (Fig. 3a). For the series of constructs expressing NS3-5A from ORF1, only the one carrying S2208I showed robust replication, whereas replication of constructs carrying all other mutations (G1911A, P2008A, and K1240N) was absent. When ORF1 expressed NS3-5B instead, the relative replication of the construct with S2208I in ORF2 was enhanced compared to its wt counterpart, but more noticeable were the differences in replication for those constructs carrying the other mutations in ORF2. Constructs with P2008A and G1911A mutations replicated as well as had been observed for wt^NS3-5B^/GDD>GAA^NS3-5B^. In theory, this was consistent with the ability of ORF1 NS3-5B simply substituting for the functional replicase produced from ORF2 NS3-5B. However, the same phenomenon was not observed for the K1240N mutation, as replication of this construct was markedly impaired, with luciferase activity barely detected even at 72 and 96 h posttransfection. Thus, while replication of intragenomic constructs carrying functional NS3-5B in ORF1 and defective NS3-5B in ORF2 might in part depend on a replacement of the ORF2 replicase by that encoded by ORF1, polyprotein mixing and composite RC formation appeared to occur also.

To compare how levels of rescue might differ using *trans*-complementation, a stable replicon cell line expressing functional NS3-5B was transfected with a bicistronic subgenomic replicon encoding a firefly luciferase reporter in ORF1 and a functional or mutated (NS3^K1240N^, NS4B^G1911A^, NS5A^P2008A^, NS5A^S2208I^, and NS5B^GDD>GAA^) NS3-5B polyprotein in ORF2. Consistent with reports from other studies, robust rescue of only NS5A^S2208I^ was observed, with levels of luciferase activity being approximately 10% of those levels seen for the functional control construct (Fig. 3b). Other mutations were rescued either at a low level (NS4B^G1911A^ and NS5A^P2008A^) or not at all (NS3^K1240N^ and NS5B^GDD>GAA^). As expected, none of the mutated transcripts replicated when transfected into naive Huh7.5 cells (Fig. 3c).

### NS3 helicase mutation K1240N is dominant negative.

**FIG 4:**
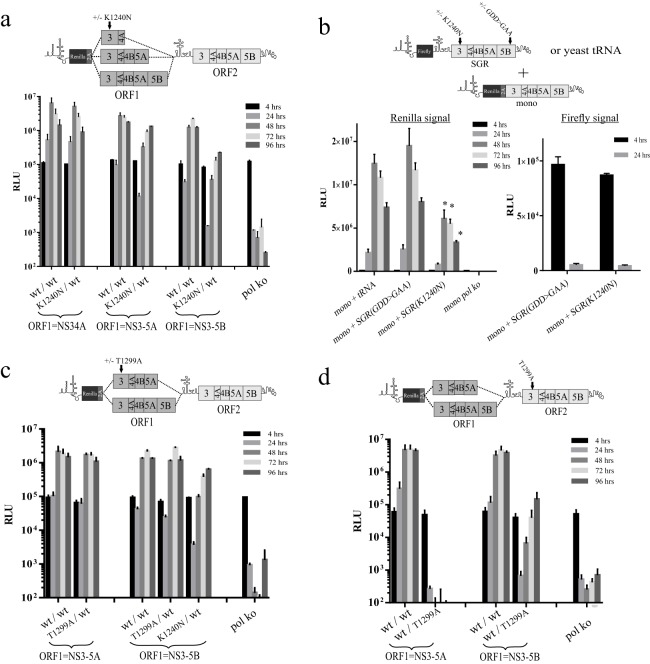
Impact of single helicase point mutations K1240N and T1299A on replicon replication. Schematics above the graphs provide details of the constructs used and the positioning of mutations within them. Replication assays either employed a single intragenomic replicon construct per experimental group transfected into Huh7.5 cells (a, c, d) or involved cotransfection of two separate RNAs into naive Huh7.5 cells to assess whether K1240N was dominant negative in *trans* (b). Results shown from the latter assay also include the firefly signal at 4 and 24 h derived from the replication-defective constructs used. Data represent the means ± SD (a, c, d) or means ± SEM (b) from 2 and 3 independent experiments, respectively. Where *n* = 3, values significantly different from the control group (mono + tRNA) are highlighted with an asterisk (*P* < 0.05; 2-tailed *t* test). Note: the assay performed to obtain the data in panel d was run in parallel with another described in the legend to Fig. 8e, and so they share control groups.

The inability to rescue the NS3 K1240N mutation in ORF2 using constructs in which ORF1 expressed wt NS3-5B was potentially consistent with the formation of composite NS protein complexes where the mutation in the NS3 helicase had a dominant negative effect on replication. To test this, the K1240N mutation was introduced into ORF1 of the intragenomic replicon carrying a functional NS3-5B in ORF2 and the impact on replication was assessed (Fig. 4a). To maximize the utility of the intragenomic system, the constructs examined included those expressing NS34A, NS3-5A, and NS3-5B from ORF1, with these polyproteins containing both wt sequences and the K1240N mutation.

From monitoring luciferase levels in cells transfected with the various constructs, K1240N^NS34A^/wt^NS3-5B^ had levels of replication comparable to those of its counterpart expressing functional NS34A from ORF1 (Fig. 4a). In contrast, expressing the K1240N mutation from NS3-5A ORF1 (K1240N^NS3-5A^/wt^NS3-5B^) gave a noticeable drop in replication of approximately 10-fold in the first 48 h compared to wt^NS3-5A^/wt^NS3-5B^, although replication recovered to some extent at later time points. The impact of expressing K1240N from the NS3-5B ORF1 was even greater, with replication levels ∼20- to 30-fold lower than those of the comparable control construct during the first 48 h and remaining low over the course of 96 h.

To establish whether a K1240N mutated polyprotein could suppress replication when expressed from a separate RNA, JFH1-mono was cotransfected into Huh7.5 cells with either yeast tRNA or bicistronic subgenomic replicons carrying a firefly luciferase reporter gene in ORF1 and K1240N- or GDD>GAA-mutated NS3-5B in ORF2 (Fig. 4b). Monitoring firefly luciferase activities in the cells 4 h and 24 h posttransfection showed that the two bicistronic replication-defective transcripts were present at broadly similar levels and exhibited similar rates of decay. Despite this, the replication of JFH1-mono was significantly inhibited in the presence of K1240N NS3-5B (replication was ∼30 to 55% of that of the tRNA group over 24 to 96 h). In contrast, inhibition was not seen in the presence of GDD>GAA-mutated NS3-5B, indicating that reduced replication imposed by K1240N was not due to competition between NS proteins for limited host cell factors (24).

### Intragenomic replicon replication of constructs carrying the T1299A helicase mutation differs from those carrying the K1240N mutation.

Kazakov et al. (18) recently proposed NS3 helicase activity to be *cis*-acting on the basis that several mutations blocking NS3 helicase activity could not be *trans*-complemented using various systems expressing NS3-5B. However, the fact that K1240N imposed a *trans*-dominant negative phenotype on replication offered a potential alternative explanation as to why rescue of helicase mutations had not been observed. It was therefore important to examine whether another mutation, T1299A, which disrupts helicase binding to the phosphate backbone of nucleic acid and used in the aforementioned study, might also be dominant negative. Hence, T1299A was introduced into ORF1 in a series of intragenomic replicons, and the impact it had on ORF2 replicase activity was assessed by comparing the replication of these constructs with that of their counterparts expressing functional polyprotein from ORF1. Remarkably, replication was unaffected by the presence of T1299A, irrespective of whether it was expressed from ORF1 NS3-5A or NS3-5B polyproteins (Fig. 4c). In contrast, a control transcript expressing K1240N from NS3-5B ORF1 did show suppressed replication.

If T1299A was not dominant negative, as our data suggested, this provided an opportunity to examine the extent to which ORF1 could replace the *cis*-acting role of ORF2. Therefore, T1299A was introduced into ORF2 of an intragenomic replicon expressing functional NS3-5A or NS3-5B from ORF1. Transfection of these transcripts into Huh7.5 cells revealed that while replication was not supported by ORF1 expressing NS3-5A, replication was detected with the construct expressing functional NS3-5B from ORF1 (Fig. 4d). We conclude that while NS3 helicase activity is likely to be a *cis*-acting function of the replicase, mutations disrupting this activity can manifest additional phenotypes, as seen with K1240N exerting a dominant negative effect on replication. We further conclude that ORF1 NS3-5B can substitute for *cis*-acting functions normally provided by the authentic NS3-5B replicase expressed from ORF2.

### Establishing that NS4B, NS5A, and NS5B mutations depend on NS3-5B for rescue.

**FIG 5:**
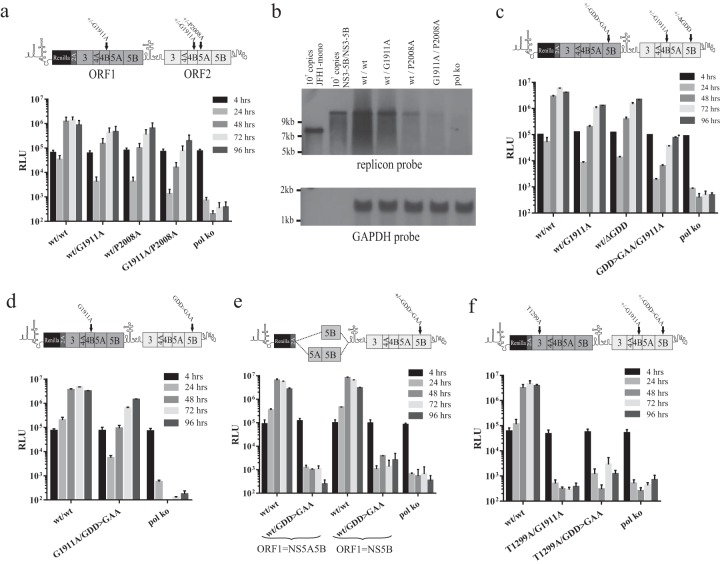
Analysis of mutations using the intragenomic replicon and assessing their dependency on NS3-5B for rescue. The selection of mutations was on the basis that they were refractile to rescue with ORF1 NS3-5A when present in ORF2 but tolerant when ORF1 instead expressed NS3-5B. These mutations were introduced into intragenomic replicons in various combinations, and replication assays were performed. Schematics above each graph (a, c, d, e, f) provide details of the constructs used and the positioning of mutations within them. Also shown are Northern blot data (b) using total cellular RNA taken from a replication assay (a). Data represent the means ± SD from two separate experiments. pol ko, polymerase knockout.

The data above indicated that NS3-5B expressed from ORF1 of an intragenomic replicon was capable of replacing the *cis*-acting functions of ORF2. To further exploit the potential of the intragenomic replicon system, mutations were introduced into wt^NS3-5B^/wt^NS3-5B^ in different combinations and permutations. Initially, we placed the NS4B G1911A mutation in ORF1 and the NS5A P2008A mutation in ORF2 to create G1911A^NS3-5B^/P2008A^NS3-5B^. RNA from this construct and other controls was transfected into Huh7.5 cells, and replication was assessed by measuring luciferase activity (Fig. 5a). The pattern of reporter activity for wt^NS3-5B^/wt^NS3-5B^, wt^NS3-5B^/G1911A^NS3-5B^, and wt^NS3-5B^/P2008A^NS3-5B^ was identical to that seen in Fig. 3. Interestingly, G1911A^NS3-5B^/P2008A^NS3-5B^ also replicated at levels that were only about 3-fold less than that for either wt^NS3-5B^/G1911A^NS3-5B^ or wt^NS3-5B^/P2008A^NS3-5B^, in contrast to GDD>GAA^NS3-5B^/ΔGDD^NS3-5B^, which did not replicate. Northern blot analysis (Fig. 5b) revealed that RNA identical in length to *in vitro*-transcribed wt^NS3-5B^/wt^NS3-5B^ transcripts was clearly present in cells 72 h after transfection with wt^NS3-5B^/wt^NS3-5B^, wt^*NS3-5*B^/G1911A^NS3-5B^, and wt^NS3-5B^/P2008A^NS3-5B^. A similar-sized band, albeit at a very reduced level, was also seen in cells transfected with G1911A^NS3-5B^/P2008A^NS3-5B^. No obvious band of this size was observed in cells transfected with the polymerase knockout control intragenomic replicon. As neither G1911A nor P2008A could be rescued by NS3-5A, we conclude that composite complexes must have formed from the mixing of two defective NS3-5B polyproteins.

Rescue of replication is proposed to occur through the exchange of NS proteins between individual polyprotein-derived complexes (18). However, published data suggest that rescue of NS5B defects instead depends on strand exchange, a process where the viral RNA transfers from an NS5B-containing complex derived from one NS3-5B ORF to another NS5B-containing complex derived from a second NS3-5B ORF (18). Under such circumstances, NS3-5B provided in *trans* is able to rescue polymerase defects only if it itself carries no lethal mutations. Our intragenomic replicon system provided an opportunity to examine the rescue of defective NS5B under conditions where two polyproteins were translated from the same RNA. Therefore, constructs in which the NS4B mutation G1911A was combined with the polymerase knockout mutation GDD>GAA were made, to give GDD>GAA^NS3-5B^/G1911A^NS3-5B^ (Fig. 5c). This construct replicated at lower levels than wt^NS3-5B^/wt^NS3-5B^ or single-mutated control constructs wt^NS3-5B^/G1911A^NS3-5B^ and wt^NS3-5B^/GDD>GAA^NS3-5B^ but still generated a luciferase signal 100-fold higher than that of the replication-defective construct GDD>GAA^NS3-5B^/ΔGDD^NS3-5B^. The two mutations were subsequently switched between ORF1 and ORF2, resulting in the generation of G1911A^NS3-5B^/GDD>GAA^NS3-5B^. Again, low but detectable levels of replication were seen in cells transfected with this replicon (Fig. 5d). The fact that both GDD>GAA^NS3-5B^/G1911A^NS3-5B^ and G1911A^NS3-5B^/GDD>GAA^NS3-5B^ replicate further supports the view that ORF1 and ORF2 within the intragenomic replicon are each capable of contributing replicase *cis*-acting functions. To confirm that rescue of a polymerase defect in *cis* required NS5B to be expressed in the context of a polyprotein, intragenomic replicons were made expressing NS5B or NS5A5B in ORF1 and encoding either a functional or polymerase-defective replicase in ORF2 (Fig. 5e). Upon transfection into Huh7.5 cells, only those constructs encoding a functional NS3-5B in ORF2 replicated, indicating that rescue of polymerase activity is indeed dependent on expression of functional NS5B in the context of a polyprotein, most probably NS3-5B.

Having established that G1911A and GDD>GAA were dependent on NS3-5B for rescue, we combined these mutations in ORF2 with the helicase mutation T1299A in ORF1. Interestingly, transfection of the resulting transcripts into Huh7.5 cells revealed that neither combination of mutations supported intragenomic replicon replication (Fig. 5f). Thus, despite evidence that composite replication complexes are formed between NS3-5B expressed from both ORFs, it appears that neither G1911A- nor GDD>GAA-mutated NS3-5B can substitute for the *cis*-acting role of a T1299A mutated polyprotein. The implications of this are discussed later, but these data suggest that each NS3-5B polyprotein that contributes to functional NS3-5B-dependent complex formation requires helicase activity.

Overall, our results show that complementation of some mutations in the NS3-5A region is indeed dependent on NS3-5B. The data also further support the notion that ORF1 can substitute for ORF2 *cis*-acting interactions and that helicase activity provided both in *cis* and in *trans* is critical for NS3-5B-dependent complex formation.

### Lethal mutations in NS3 and NS4A that can be rescued in the intragenomic system exist.

The above data indicated that all NS3 mutations that blocked helicase activity were refractile to complementation. To determine whether this was representative of all mutations in NS3 and its cofactor NS4A, we selected four further mutations in these proteins that were lethal to replication but did not cause defective polyprotein processing (25–28). Two mutations were in NS3, mapping to the protease domain (L1157A) and hinge region between the protease and helicase domains (PP1220-1GG), respectively. The other two mutations were situated at either end of NS4A (V1665G and Y1706A), the former locating to and disrupting NS4A transmembrane dimerization and the latter representing an essential conserved residue within the acidic domain of NS4A.

**FIG 6:**
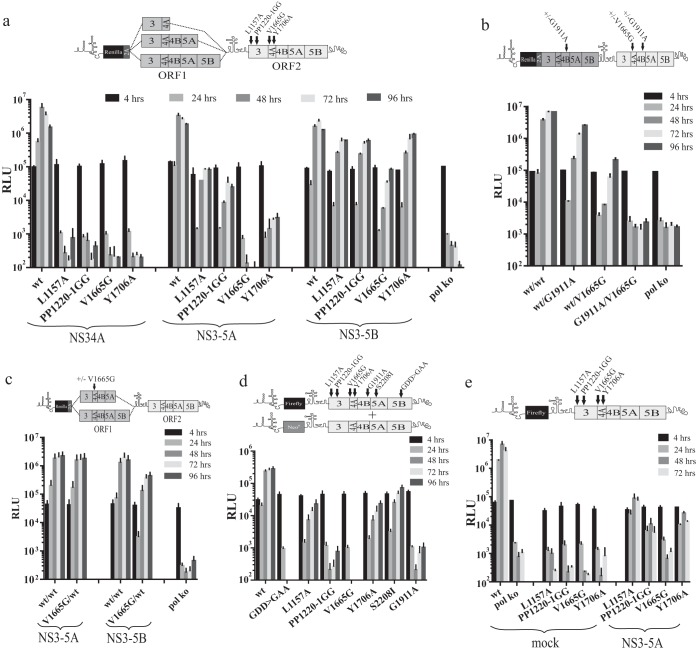
Rescue of lethal mutations in NS3 and NS4A that disrupt processes other than NS3 helicase activity. Genetic complementation of genetic defects was assessed in a variety of ways, including use of intragenomic replicons transfected into Huh7.5 cells (a, c) or using bicistronic replicon expressing firefly luciferase from ORF1 transfected into either a helper replicon cell line (d) or into naive Huh7.5 cells transduced with either baculovirus expressing NS3-5A or β-galactosidase (mock control) (e). Intragenomic replicons were also used to assess whether V1665G, which appeared refractile to rescue with NS3-5B, was dominant negative (c). Schematics above each graph provide specific details of the constructs used and the positioning of mutations within them. Data represent the means ± SD from two separate experiments.

The mutations were introduced into ORF2 of intragenomic replicons that expressed NS34A, NS3-5A, or NS3-5B from ORF1. All constructs expressing NS34A were replication defective (Fig. 6a). In contrast, three of the constructs (L1157A, PP1220-1GG, and Y1706A) replicated when ORF1 expressed NS3-5A instead; luciferase values indicated that L1157A was rescued most efficiently, followed by PP1220-1GG and then Y1706A. When ORF1 expressed NS3-5B, replication occurred for all four mutations. For L1157A, PP1220-1GG, and Y1706A, levels of replication were broadly comparable, while they were lower but still detectable for V1665G. To establish whether the latter mutation was rescuable in an NS3-5B-dependent manner, the mutation was introduced into ORF2 of an intragenomic construct expressing an NS4B G1911A-mutated NS3-5B from ORF1. For comparison, constructs expressing wt NS3-5B from ORF1 and V1665G- or G1911A-mutated NS3-5B from ORF2 were included. Transfection into Huh7.5 cells revealed that for those constructs carrying only a single mutation in ORF2, V1665G had a more inhibitory effect on replication than did G1911A (Fig. 6b). The replicon carrying G1911A in ORF1 and V1665G in ORF2 did not replicate. The fact that wt^NS3-5B^/V1665G^NS3-5B^ replicated at a noticeably lower level than wt^NS3-5B^/G1911A^NS3-5B^ suggested that V1665G might exert a dominant negative phenotype. To establish whether this was the case, the mutation was introduced into NS3-5A or NS3-5B expressed from ORF1 (Fig. 6c), and the replication of these intragenomic constructs was compared to the equivalent constructs expressing functional NS3-5A and NS3-5B from ORF1. Interestingly, the presence of V1665G in ORF1 suppressed replication compared to equivalent constructs expressing functional polyprotein, but only when ORF1 encoded NS3-5B. It therefore appears that V1665G is dominant negative but, unlike the K1240N NS3 helicase mutation, manifests this phenotype only when expressed in the context of a full-length replicase.

To compare the levels of rescue with that seen using *trans*-complementation, all four mutations were introduced into a bicistronic subgenomic replicon encoding a firefly luciferase reporter in ORF1. A stable cell line expressing a functional replicon was transfected with these constructs along with control constructs carrying the GDD>GAA, G1911A, and S2208I mutations to allow comparisons to be made with earlier experiments (Fig. 6d). Other than GDD>GAA and V1665G, all mutations showed some degree of complementation. For the NS3 mutation L1157A and NS4A mutation Y1706A, levels of rescue were only slightly less than that observed for the NS5A mutation S2208I. In contrast, rescue of the other NS3-5A dependent mutation in NS3, PP1220-1GG, was lower and comparable to that seen for the NS3-5B-dependent mutation G1911A in NS4B. None of the mutated constructs replicated when transfected into naive Huh7.5 cells (data not shown). It was interesting that Y1706A was rescued as efficiently as L1157A given the limited extent to which the former mutation was rescued by NS3-5A when expressed from ORF1 of an intragenomic replicon. We therefore transfected the same firefly replicon constructs into Huh7.5 cells transduced with baculovirus expressing NS3-5A (Fig. 6e). While the data confirmed that rescue of L1157A, PP1220-1GG, and Y1706A required expression of a minimum NS3-5A polyprotein, these levels were reasonably comparable for L1157A and Y1706A, similar to the situation using helper replicons.

We conclude that as in NS5A, there are mutations in NS34A capable of being rescued by a minimum NS3-5A precursor whereas others are not. The rescue of mutations dependent on a minimum NS3-5A polyprotein is possible when this protein is expressed in *cis* from ORF1 or in *trans* from a separate mRNA.

### Blocking NS4B5A cleavage impacts only on functions within complexes dependent on NS3-5B expression.

Since cleavage between NS4B and NS5A by NS3/4A is slower than at other boundary sites in the NS3-5B polyprotein, we and others have proposed that the presence of an NS4B5A precursor within a complex destined to form the RC might serve as a checkpoint to prevent premature membrane deformation events linked to RC maturation (29, 30). Given our data suggesting that NS3-5A and NS3-5B provide differential access to different NS protein complexes supporting replication, we were interested to know the impact that blocking cleavage of NS4B5A would have in the context of the intragenomic replicon system.

**FIG 7:**
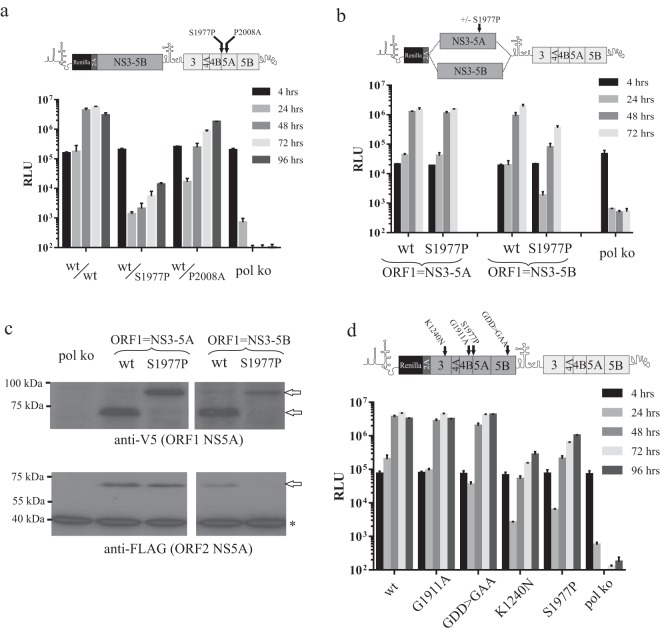
Effects of blocking NS4B5A boundary cleavage on intragenomic replication. Schematics provide details of the constructs used and the positioning of mutations within them. Cleavage of the NS4B5A boundary was blocked by the S1977P mutation. The impact that this had on intragenomic replicons when it was introduced into ORF2 (a) and ORF1 (b) is shown, with cell lysates taken from the latter experiment 72 h posttransfection also analyzed by Western blotting (c). Arrows indicate the position of NS5A and the uncleaved NS4B5A precursor. An asterisk indicates the position of a cross-reacting cellular protein detected by the anti-FLAG antibody. Data from a replication assay comparing the abilities of different mutated NS3-5B ORF1s, including that of S1977P, to suppress replication of intragenomic replicons carrying functional ORF2 (d) are also provided. Data represent the means ± SD from two separate experiments. Note: the assay performed to obtain data in panel b was run in parallel with that described in Fig. 8b, and so the two share control groups.

In the first instance, an NS5A mutation, S1977P, designed to block NS4B5A boundary cleavage, was introduced into ORF2 of an intragenomic replicon expressing NS3-5B in ORF1. Transfection of this construct into Huh7.5 cells showed that, compared to a control construct carrying the NS5A P2008A mutation in ORF2, replication was considerably impaired (Fig. 7a). S1977P was then introduced into NS3-5A or NS3-5B expressed from ORF1 of an intragenomic replicon (Fig. 7b). As was observed for the K1240N and V1665G mutations, NS3-5B expressed from ORF1 carrying the S1977P mutation measurably impaired replication (Fig. 7b). Similar to the V1665G mutation but unlike the K1240N mutation, S1977P failed to inhibit replication when expressed from ORF1 in the context of NS3-5A. Western blot analysis confirmed the presence of uncleaved NS4B5A from ORF1 in cell extracts from cells transfected with S1977P^NS3-5A^/wt^NS3-5B^ and S1977P^NS3-5B^/wt^NS3-5B^ (Fig. 7c). These data suggested that S1977P had dominant negative activity, albeit only when expressed in the context of NS3-5B. This was verified by comparing the impact that K1240N, S1977P, G1911A, and GDD>GAA mutations in ORF1 had on replication (Fig. 7d). Critically, only K1240N and S1977P markedly suppressed replication, while the other mutations exhibited little or no effect.

**FIG 8:**
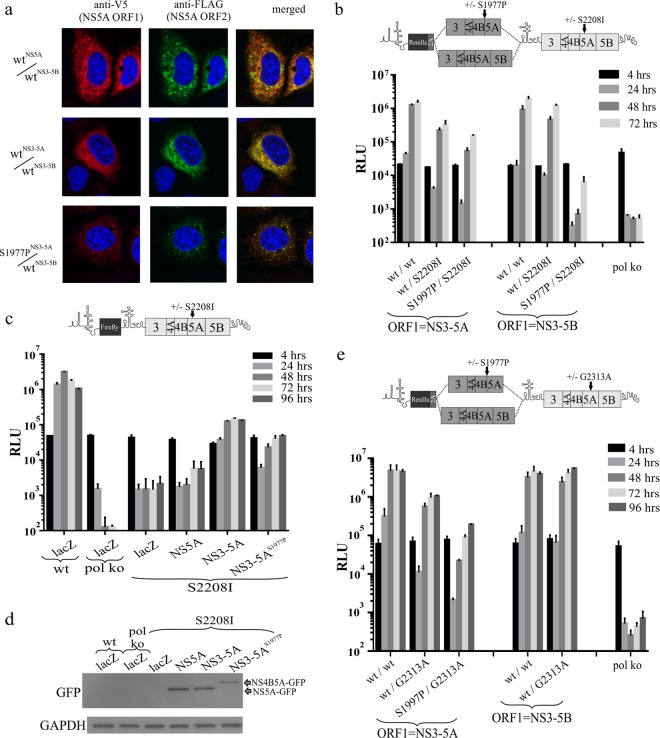
Impact of blocking NS4B5A cleavage on NS3-5A function. (a) Intragenomic replicons with a functional NS3-5B encoded in ORF2 and expressing either NS5A, NS3-5A, or an S1977P-mutated NS3-5A from ORF1 were transfected into Huh7.5 cells. Confocal microscopy visualized the extent of ORF1 V5-tagged NS5A and ORF2 FLAG-tagged NS5A colocalization 72 h posttransfection. (b and e) Intragenomic replicon replication assays detail the ability of an S1977P-mutated polyprotein expressed from ORF1 to complement either the NS5A S2208I (b) or NS5A G2313A (e) defect in ORF2, the schematics above the graphs indicating further details of the constructs used. (c) *Trans*-complementation of a firefly luciferase-expressing bicistronic replicon carrying the S2208I mutation is assessed using Huh7.5 cells transduced with baculovirus expressing either β-galactosidase (*lacZ*) or GFP-tagged versions of NS5A, NS3-5A, and NS3-5A carrying the S1977P mutation. Western blot analysis of cell lysates taken 72 h posttransfection shows levels of baculovirus-transduced expression (d). Graphical data represent the means ± SD from two separate experiments.

Two possible explanations might account for the failure of NS3-5A carrying the NS5A^S1977P^ mutation to inhibit replication. The first was that complexes formed by NS3-5A tolerated the presence of an uncleaved NS4B5A precursor, unlike those formed by NS3-5B. The second possibility was that the mutation had prevented integration of NS4B5A into NS3-5A-dependent complexes. As the latter event might have allowed dissociation of NS4B5A from the MAF, confocal analysis was employed to examine the location of the NS4B5A precursor from S1977P^NS3-5A^/wt^NS3-5B^ with respect to the functional NS5A expressed from ORF2. Results showed that extensive overlap of signals was evident (Fig. 8a).

To further investigate the impact of S1997P on NS3-5A-dependent complex formation, we took advantage of our earlier observation that rescue of the NS5A S2208I mutation occurred extremely efficiently using NS3-5A, reasoning that if S1977P NS3-5A still formed NS3-5A-dependent complexes, it might be able to rescue the latter mutation. Intragenomic constructs carrying S1977P-mutated NS3-5A and NS3-5B in ORF1 were therefore further modified to carry the S2208I mutation in ORF2. Remarkably, despite being unable to produce mature NS5A, an NS3-5A polyprotein carrying S1977P expressed from ORF1 could rescue replication when ORF2 contained the S2208I mutation (Fig. 8b), albeit 3- to 4-fold less efficiently than when ORF1 expressed functional NS3-5A. In contrast, the construct expressing S1977P-mutated NS3-5B from ORF1 and S2208I-mutated NS3-5B in ORF2 barely replicated. To verify this result, *trans*-complementation of the S2208I mutation was examined under conditions where functional NS5A, functional NS3-5A, and S1977P-mutated NS3-5A were provided by baculovirus transduction (Fig. 8c). While wt NS3-5A was most effective at rescuing the S2208I mutation, expression of S1977P-mutated NS3-5A also resulted in a substantial level of rescue that was severalfold higher than that seen using NS5A alone. Western blot analysis (Fig. 8d) established that NS5A expression was greatest in NS5A- and lowest in NS3-5A^S1977P^-transduced cells, indicating that expression levels do not account for the improved rescue of S2208I with NS3-5A^S1977P^ versus NS5A alone.

To extend our analysis to another defect in NS5A, we selected a further mutation (G2313A) belonging to an intragenic complementation group different from that of S2208I and P2008A (17). This mutation was introduced into ORF2 of a series of intragenomic constructs expressing either wt NS3-5A, S1977P-mutated NS3-5A, or wt NS3-5B from ORF1. Transfection of the RNAs into Huh7.5 cells revealed that NS3-5A was able to efficiently rescue G2313A and that this still occurred, albeit at slightly lower levels, when ORF1 instead carried an S1977P-mutated NS3-5A (Fig. 8e). On the basis of these combined results, we conclude that at least some NS5A functions operate within an NS3-5A-dependent complex that is physically distinct from that formed by NS3-5B/NS3-5B interactions.

## DISCUSSION

Our results demonstrate that the truncated polyprotein NS3-5A, although able to target NS protein components to MAF, can complement only certain replication defects contained in the same coding region. As replication depends on homo- and heterotypic interactions between different NS proteins within NS3-5B (18, 31–36), functional NS-NS protein complex formation would explain this observation. Our proposal is that the inability to rescue certain mutations by NS3-5A is due to this precursor participating in the formation of only a subset of complexes formed by NS3-5B/NS3-5B interactions. Evidence supporting this conclusion comes from several observations. First, two mutations in the NS3-5A coding region were not complemented by NS3-5A but were efficiently rescued by NS3-5B. While this rescue, in theory, could be due to NS3-5A being less efficient at forming the same complexes as NS3-5B, experimental data suggest otherwise. We identified one mutation (PP1220-1GG) whose defect was rescued by NS3-5A, yet the same mutation showed comparable rescue to the NS3-5B-dependent defects G1911A and P2008A using helper virus. Second, despite the fact that the three dominant mutations, K1240N, V1665G, and S1977P, inhibited replication to broadly similar levels when expressed in the context of NS3-5B, only K1240N inhibited replication when expressed as an NS3-5A precursor. Finally, expression of the dominant negative mutation, S1977P, in the context of an NS3-5A precursor enabled rescue of two NS5A mutations belonging to different intragenic groupings. Given that all three mutations are on the same protein, it seems probable that S1977P expressed from NS3-5A must be selectively incorporated into an NS5A-containing complex disabled by either S2208I or G2313A, while being excluded from another complex for which S1977P itself is lethal.

Rescue of replication-defective mutations in positive-strand RNA viruses has typically employed *trans*-complementation systems, where the functional protein or polyprotein is expressed from an RNA separate from the one being rescued. While our study is not the first to produce two copies of an NS protein from a replication-competent RNA, it is the first to express an entire duplicated replicase. The use of similar constructs has proven problematic in the past. Rescue experiments that relied on the placement of duplicated NS coding regions in poliovirus found that the duplicated sequence was rapidly lost (37), presumably because of selective pressures arising from the use of infectious virus with larger-than-unit-sized genomes and the subsequent packaging constraints that this imposed. In addition, the use of virus rather than replicons meant that these selection pressures operated across an entire cell population rather than within individual transfected cells. By using replicons carrying the second copy of the NS polyprotein as a recoded sequence, we have studied replication under transient conditions where recombination, if it occurs, makes little or no contribution to the overall assay readout. Supporting this view, we found that constructs expressing functional NS3-5A from ORF1 and NS3-5B from ORF2 failed to generate a replication signal when ORF2 carried mutations K1240N, V1665G, G1911A, and P2008A, despite the fact that a single recombination event had the potential to generate replication-competent monomeric transcripts. Northern blot analysis of replication assays 72 h posttransfection also showed that the replicon transcripts present in cells were the same size as the input transcripts. The tight temporal and spatial control of expression obtained from producing the two replicases from the same RNA makes the intragenomic replicon system a potentially valuable tool for studying replication of other positive-strand RNA viruses. That NS3-5B expressed from either ORF is able to assume *cis*-acting roles in replication adds further value to the system, as it provides opportunities to look at replication complex formation in ways that have not been possible until now.

RNA replication depends not only on viral polyprotein expression but also on the interactions between these polyprotein components and RNA elements present in the genome. In HCV, the NS3-5B coding region lies adjacent to the 3′ UTR and contains *cis*-acting RNA elements within its 3′ end that make essential kissing loop interactions (22, 23, 38). Remarkably, there is no requirement for the NS3-5B coding region to overlap the kissing loop structure (38), although until now it has been unclear whether there is a spatial relationship between the NS3-5B ORF and the 3′ end of the genome. By demonstrating that NS3-5B expressed from ORF1 of an intragenomic replicon can support replication when ORF2 carries the *cis*-acting NS3 helicase mutation T1299A (18), our data indicate that a considerable distance between the polyprotein ORF and the 3′ UTR can be tolerated. This conclusion is further supported by our finding that intragenomic constructs expressing two NS3-5B polyproteins, each with separate mutations in NS4B (G1911A) or NS5B (GDD>GAA), still replicate, irrespective of which ORF the two mutations are placed in. Based on studies by others (18), such combinations would not be tolerated if ORF1 did not assume *cis*-acting functions normally provided by the authentic replicase encoded by ORF2.

**FIG 9:**
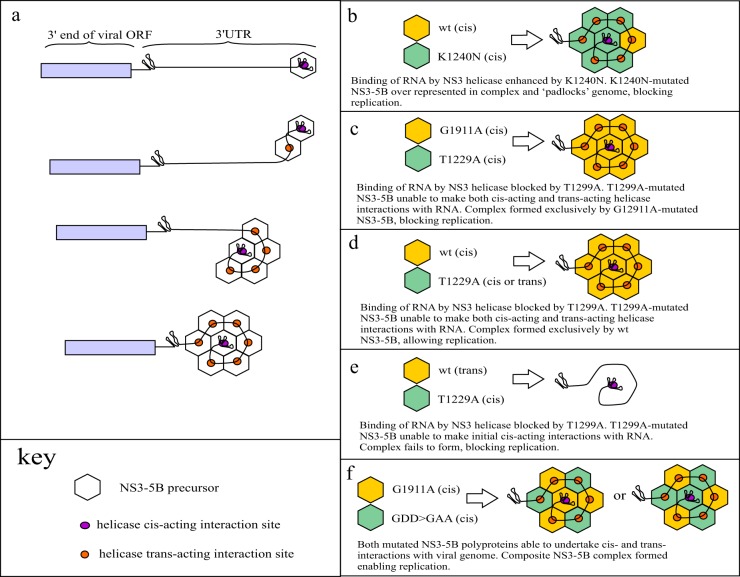
Putative model of NS3-5B-dependent complex assembly. (a) The NS3 helicase domain within a recently translated polyprotein precursors binds in a *cis*-dependent manner to the viral genome (for illustrative purposes, the interaction is shown to occur within the 3′-UTR X region). Subsequent recruitment of additional copies of NS3-5B precursor is not a *cis*-acting event but does depend on them possessing both NS5B and helicase-dependent RNA binding activity. Our data showing that blocking of NS4B5A cleavage creates a dominant negative polyprotein precursor suggest that while complex activity requires completion of polyprotein proteolytic processing, complex assembly does not. (b to f) The outcome of NS3-5B-dependent complex formation is shown using the same criteria as described above. Replication requires that (i) complex assembly occurs, (ii) any mutated NS protein present is rescued by a functional counterpart, and (iii) a polyprotein carrying a dominant negative mutation is not recruited.

As our data showed that ORF1 is able to substitute for the *cis*-acting role of ORF2, it was surprising to see that intragenomic constructs carrying the *cis*-acting NS3 helicase mutation T1299A in ORF1 were unable to tolerate the presence of the NS4B G1911A mutation in ORF2. This was despite the fact that neither of these mutations was dominant negative and data from both this study and others showing that the function blocked by G1911A is not *cis*-acting (14). Given that NS3-5A expressed from ORF1 of an intragenomic replicon cannot rescue T1299A in ORF2 and that G1911A requires NS3-5B for rescue, it seems likely that both *cis*-acting and *trans*-acting events blocked by these two mutations localize to an NS3-5B-containing complex from which NS3-5A is excluded. Based on this conclusion and other observations, we propose the following model, in which formation of this complex involves an association of multiple copies of an NS3-5B precursor shortly after their translation. First, polyprotein association is catalyzed by an initial binding event between a *cis*-acting replication element (CRE) within the RNA genome and NS3 helicase, as has been suggested by others (18). However, subsequent assembly requires the presence of NS5B as a structural component, both in the polyprotein that makes the *cis*-acting contact with the viral RNA and in the polyproteins that engage in *trans* with this initial *cis*-acting complex. This requirement for NS5B has similarities to the model put forward by Kazakov et al. (18), who suggested that the protein provided a structural component for *cis*-dependent interactions but did not identify a similar role for it in *trans*. A further distinguishing feature of our model is that incorporation of NS3-5B into this growing complex also requires the newly arriving polyproteins to display NS3 helicase RNA binding activity. In other words, NS3 helicase has both essential *cis*- and *trans*-acting roles in NS3-5B-dependent complex formation. Figure 9 illustrates this as well as how the assembly of such a complex would be influenced by the different mutations used in this study. Importantly, some of the predictions made by our model can account for the findings of others. It has been shown that replication of viral transcripts expressing NS3-5A in *cis* cannot be supported by expression of NS3-5B in *trans* (18), despite the fact that replication of minigenomes that fail to express viral proteins can be supported by expression of a viral replicase (39). Our model offers the possibility that NS3-5A expressed in *cis* is capable of making nonproductive interactions with CREs in the viral genome, thus frustrating the assembly of the NS3-5B-dependent complex. Perhaps relating to this mode of action, we identified one mutation, NS4A^Y1706A^, that was rescued far more effectively when NS3-5A was expressed in *trans* from a separate RNA than in *cis* when expressed from ORF1 of an intragenomic replicon. Identifying mutations in ORF2 NS3-5B that weaken its ability to outcompete ORF1-expressed NS3-5A for binding to CREs could provide useful information about protein-protein and protein-RNA contacts made during complex assembly. While the NS4A^Y1706^ residue is relatively conserved, it does lie adjacent to another more-variable residue (position 1708 in JFH1) previously implicated in orchestrating physical interactions between NS4A and all other NS proteins within the NS3-5B polyprotein (32). It would be interesting to see whether mutations of residues such as those identified by covariant amino acid analysis alter the ability of different polyproteins expressed in *cis* to compete for CREs.

A general feature of the mutations dependent on NS3-5A for rescue compared to those dependent on NS3-5B was the greater extent with which they were *trans*-complemented by a helper replicon. The reduced levels of stringency for rescue fit with a proposal by Fridell and colleagues that NS5A supports functions both inside and outside the replication complex (16). Importantly, they speculated that mutations blocking NS5A function outside and inside the replication complex included S2208I and P2008A; these mutations disrupted NS3-5A- and NS3-5B-dependent complex activity, respectively, in our hands. Compartmentalization of NS3-5A- and NS3-5B-dependent complex functions is certainly a possibility that warrants consideration. Assuming that exchange of individual NS proteins within complexes occurs (18), without compartmentalization it is difficult to envisage why some mutations in the NS3-5A region show dependence on NS3-5B for rescue whereas others in the same NS protein are efficiently rescued by NS3-5A. Furthermore, the proposed role that cleavage of the NS4B5A boundary has in enabling NS5A-driven double-membrane formation (29, 30), coupled to the fact that the S1977P mutation, which blocks boundary cleavage, is only dominant negative as an NS3-5B precursor, is consistent with NS3-5A-dependent complexes being excluded from the RC. Our attempts to see whether discrete compartmentalization occurs have so far been restricted to confocal microscopy analysis. Given the size of the double-membrane vesicles in MAF, the structures thought to contain the functional replication complexes, it is perhaps not surprising that we failed to observe separation of NS3-5A- versus NS3-5B-dependent complexes (data not shown). Further physical and electron microscopy studies are planned.

An unexpected outcome of our study was the *trans*-dominant negative phenotype displayed by one NS3 helicase mutation (K1240N) but not by another (T1299A). While both mutations block helicase activity, this occurs through disruption of different sites within the enzyme. NS3^K1240N^ disrupts the Walker A motif, responsible and required for ATP binding and hydrolysis, whereas NS3^T1299A^ blocks RNA helicase binding by disrupting the TxGx motif, involved in phosphate backbone interactions (40). Disruption of the Walker A motif would also have the likely consequence of locking the enzyme in an ATP-free open state, potentially increasing its propensity to bind RNA compared to wild-type NS3 (41). Thus, one possible reason for NS3^K1240N^ displaying a dominant negative phenotype is that it displays either a normal or enhanced ability to bind to both CREs and other RNA sites on the RNA genome, ensuring that defective NS3 populates NS3-5B-dependent complexes. However, the fact that NS3^K1240N^ was also dominant negative when expressed as an NS3-5A precursor suggests that the inhibitory effect extends beyond the NS3-5B-dependent complex. Perhaps the mutated NS3 helicase “padlocks” itself onto viral RNA present within the membranous web, immobilizing it and/or preventing functional helicase molecules translocating along it. Understanding what mechanisms may be involved is important. Studies have already highlighted the potential importance of *trans*-dominant mutations in guiding the development of novel viral inhibitors (42). Should pharmacological recapitulation of this dominant negative phenotype prove possible, it would provide added therapeutic benefit to any drug targeting helicase activity.

In summary, our work reveals the existence of two functional sets of complexes that reside within the HCV MAF, both of which are necessary for replication of the viral genome and each of which performs distinct functions. It seems unlikely that this is a unique mechanism adopted by HCV. Rescue of defects in pestivirus NS5A shows remarkable similarities to that seen for HCV NS5A, with some but not all mutations being readily rescued by helper virus constructs (43). Similarly, certain defects in flavivirus NS proteins can be complemented, but other defects in the same protein cannot (44). The intragenomic replicon system represents a potential powerful approach to investigate the formation and function of complexes, in part due to its ability to overcome the traditional *cis*-acting barriers encountered by *trans*-complementation. Understanding the roles that different complexes play will inform general aspects of viral RNA replication.

## Supplementary Material

Supplemental material

## References

[1] Feeney ER, Chung RT 2014 Antiviral treatment of hepatitis C. BMJ 348:g3308. doi:10.1136/bmj.g3308. 25002352 PMC6880951

[2] Scheel TK, Rice CM 2013 Understanding the hepatitis C virus life cycle paves the way for highly effective therapies. Nat Med 19:837–849. doi:10.1038/nm.3248. 23836234 PMC3984536

[3] Tsukiyama-Kohara K, Iizuka N, Kohara M, Nomoto A 1992 Internal ribosome entry site within hepatitis C virus RNA. J Virol 66:1476–1483. 1310759 10.1128/jvi.66.3.1476-1483.1992 PMC240872

[4] Lohmann V, Korner F, Koch J, Herian U, Theilmann L, Bartenschlager R 1999 Replication of subgenomic hepatitis C virus RNAs in a hepatoma cell line. Science 285:110–113. doi:10.1126/science.285.5424.110. 10390360

[5] Raney KD, Sharma SD, Moustafa IM, Cameron CE 2010 Hepatitis C virus non-structural protein 3 (HCV NS3): a multifunctional antiviral target. J Biol Chem 285:22725–22731. doi:10.1074/jbc.R110.125294. 20457607 PMC2906261

[6] Behrens SE, Tomei L, De FR 1996 Identification and properties of the RNA-dependent RNA polymerase of hepatitis C virus. EMBO J 15:12–22. 8598194 PMC449913

[7] Einav S, Gerber D, Bryson PD, Sklan EH, Elazar M, Maerkl SJ, Glenn JS, Quake SR 2008 Discovery of a hepatitis C target and its pharmacological inhibitors by microfluidic affinity analysis. Nat Biotechnol 26:1019–1027. doi:10.1038/nbt.1490. 18758449 PMC4110250

[8] Foster TL, Belyaeva T, Stonehouse NJ, Pearson AR, Harris M 2010 All three domains of the hepatitis C virus nonstructural NS5A protein contribute to RNA binding. J Virol 84:9267–9277. doi:10.1128/JVI.00616-10. 20592076 PMC2937630

[9] Gosert R, Egger D, Lohmann V, Bartenschlager R, Blum HE, Bienz K, Moradpour D 2003 Identification of the hepatitis C virus RNA replication complex in Huh-7 cells harboring subgenomic replicons. J Virol 77:5487–5492. doi:10.1128/JVI.77.9.5487-5492.2003. 12692249 PMC153965

[10] Romero-Brey I, Merz A, Chiramel A, Lee JY, Chlanda P, Haselman U, Santarella-Mellwig R, Habermann A, Hoppe S, Kallis S, Walther P, Antony C, Krijnse-Locker J, Bartenschlager R 2012 Three-dimensional architecture and biogenesis of membrane structures associated with hepatitis C virus replication. PLoS Pathog 8:e1003056. doi:10.1371/journal.ppat.1003056. 23236278 PMC3516559

[11] Paul D, Hoppe S, Saher G, Krijnse-Locker J, Bartenschlager R 2013 Morphological and biochemical characterization of the membranous hepatitis C virus replication compartment. J Virol 87:10612–10627. doi:10.1128/JVI.01370-13. 23885072 PMC3807400

[12] Berger C, Romero-Brey I, Radujkovic D, Terreux R, Zayas M, Paul D, Harak C, Hoppe S, Gao M, Penin F, Lohmann V, Bartenschlager R 2014 Daclatasvir-like inhibitors of NS5A block early biogenesis of hepatitis C virus-induced membranous replication factories, independent of RNA replication. Gastroenterology 147:1094–1105. doi:10.1053/j.gastro.2014.07.019. 25046163

[13] Gouttenoire J, Montserret R, Paul D, Castillo R, Meister S, Bartenschlager R, Penin F, Moradpour D 2014 Aminoterminal amphipathic alpha-helix AH1 of hepatitis C virus nonstructural protein 4B possesses a dual role in RNA replication and virus production. PLoS Pathog 10:e1004501. doi:10.1371/journal.ppat.1004501. 25392992 PMC4231108

[14] Jones DM, Patel AH, Targett-Adams P, McLauchlan J 2009 The hepatitis C virus NS4B protein can trans-complement viral RNA replication and modulates production of infectious virus. J Virol 83:2163–2177. doi:10.1128/JVI.01885-08. 19073716 PMC2643717

[15] Madan V, Paul D, Lohmann V, Bartenschlager R 2014 Inhibition of HCV replication by cyclophilin antagonists is linked to replication fitness and occurs by inhibition of membranous web formation. Gastroenterology 146:1361–1372. doi:10.1053/j.gastro.2014.01.055. 24486951

[16] Appel N, Herian U, Bartenschlager R 2005 Efficient rescue of hepatitis C virus RNA replication by trans-complementation with nonstructural protein 5A. J Virol 79:896–909. doi:10.1128/JVI.79.2.896-909.2005. 15613318 PMC538567

[17] Fridell RA, Valera L, Qiu D, Kirk MJ, Wang C, Gao M 2013 Intragenic complementation of hepatitis C virus NS5A RNA replication-defective alleles. J Virol 87:2320–2329. doi:10.1128/JVI.02861-12. 23236071 PMC3571461

[18] Kazakov T, Yang F, Ramanathan HN, Kohlway A, Diamond MS, Lindenbach BD 2015 Hepatitis C virus RNA replication depends on specific cis- and trans-acting activities of viral nonstructural proteins. PLoS Pathog 11:e1004817. doi:10.1371/journal.ppat.1004817. 25875808 PMC4395149

[19] Herod MR, Schregel V, Hinds C, Liu M, McLauchlan J, McCormick CJ 2014 Genetic complementation of hepatitis C virus nonstructural protein functions associated with replication exhibits requirements that differ from those for virion assembly. J Virol 88:2748–2762. doi:10.1128/JVI.03588-13. 24352463 PMC3958097

[20] Adair R, Patel AH, Corless L, Griffin S, Rowlands DJ, McCormick CJ 2009 Expression of hepatitis C virus (HCV) structural proteins in trans facilitates encapsidation and transmission of HCV subgenomic RNA. J Gen Virol 90:833–842. doi:10.1099/vir.2008.006049-0. 19223490

[21] Simmonds P 2012 SSE: a nucleotide and amino acid sequence analysis platform. BMC Res Notes 5:50. doi:10.1186/1756-0500-5-50. 22264264 PMC3292810

[22] Lee H, Shin H, Wimmer E, Paul AV 2004 *cis*-acting RNA signals in the NS5B C-terminal coding sequence of the hepatitis C virus genome. J Virol 78:10865–10877. doi:10.1128/JVI.78.20.10865-10877.2004. 15452207 PMC521798

[23] You S, Stump DD, Branch AD, Rice CM 2004 A cis-acting replication element in the sequence encoding the NS5B RNA-dependent RNA polymerase is required for hepatitis C virus RNA replication. J Virol 78:1352–1366. doi:10.1128/JVI.78.3.1352-1366.2004. 14722290 PMC321395

[24] Lohmann V, Hoffmann S, Herian U, Penin F, Bartenschlager R 2003 Viral and cellular determinants of hepatitis C virus RNA replication in cell culture. J Virol 77:3007–3019. doi:10.1128/JVI.77.5.3007-3019.2003. 12584326 PMC149776

[25] Isken O, Langerwisch U, Jirasko V, Rehders D, Redecke L, Ramanathan H, Lindenbach BD, Bartenschlager R, Tautz N 2015 A conserved NS3 surface patch orchestrates NS2 protease stimulation, NS5A hyperphosphorylation and HCV genome replication. PLoS Pathog 11:e1004736. doi:10.1371/journal.ppat.1004736. 25774920 PMC4361677

[26] Kohlway A, Pirakitikulr N, Ding SC, Yang F, Luo D, Lindenbach BD, Pyle AM 2014 The linker region of NS3 plays a critical role in the replication and infectivity of hepatitis C virus. J Virol 88:10970–10974. doi:10.1128/JVI.00745-14. 24965468 PMC4178846

[27] Kohlway A, Pirakitikulr N, Barrera FN, Potapova O, Engelman DM, Pyle AM, Lindenbach BD 2014 Hepatitis C virus RNA replication and virus particle assembly require specific dimerization of the NS4A protein transmembrane domain. J Virol 88:628–642. doi:10.1128/JVI.02052-13. 24173222 PMC3911751

[28] Phan T, Kohlway A, Dimberu P, Pyle AM, Lindenbach BD 2011 The acidic domain of hepatitis C virus NS4A contributes to RNA replication and virus particle assembly. J Virol 85:1193–1204. doi:10.1128/JVI.01889-10. 21047963 PMC3020511

[29] Herod MR, Jones DM, McLauchlan J, McCormick CJ 2012 Increasing rate of cleavage at boundary between non-structural proteins 4B and 5A inhibits replication of hepatitis C virus. J Biol Chem 287:568–580. doi:10.1074/jbc.M111.311407. 22084249 PMC3249110

[30] Romero-Brey I, Berger C, Kallis S, Kolovou A, Paul D, Lohmann V, Bartenschlager R 2015 NS5A domain 1 and polyprotein cleavage kinetics are critical for induction of double-membrane vesicles associated with hepatitis C virus replication. mBio 6(4):e00759. doi:10.1128/mBio.00759-15. 26152585 PMC4488949

[31] Bartenschlager R, Lohmann V, Wilkinson T, Koch JO 1995 Complex formation between the NS3 serine-type proteinase of the hepatitis C virus and NS4A and its importance for polyprotein maturation. J Virol 69:7519–7528. 7494258 10.1128/jvi.69.12.7519-7528.1995 PMC189690

[32] Campo DS, Dimitrova Z, Mitchell RJ, Lara J, Khudyakov Y 2008 Coordinated evolution of the hepatitis C virus. Proc Natl Acad Sci U S A 105:9685–9690. doi:10.1073/pnas.0801774105. 18621679 PMC2474538

[33] Gouttenoire J, Roingeard P, Penin F, Moradpour D 2010 Amphipathic alpha-helix AH2 is a major determinant for the oligomerization of hepatitis C virus nonstructural protein 4B. J Virol 84:12529–12537. doi:10.1128/JVI.01798-10. 20926561 PMC3004355

[34] Lim PJ, Chatterji U, Cordek D, Sharma SD, Garcia-Rivera JA, Cameron CE, Lin K, Targett-Adams P, Gallay PA 2012 Correlation between NS5A dimerization and hepatitis C virus replication. J Biol Chem 287:30861–30873. doi:10.1074/jbc.M112.376822. 22801423 PMC3436329

[35] Paredes AM, Blight KJ 2008 A genetic interaction between hepatitis C virus NS4B and NS3 is important for RNA replication. J Virol 82:10671–10683. doi:10.1128/JVI.00875-08. 18715921 PMC2573200

[36] Paul D, Romero-Brey I, Gouttenoire J, Stoitsova S, Krijnse-Locker J, Moradpour D, Bartenschlager R 2011 NS4B self-interaction through conserved C-terminal elements is required for the establishment of functional hepatitis C virus replication complexes. J Virol 85:6963–6976. doi:10.1128/JVI.00502-11. 21543474 PMC3126587

[37] Cao X, Wimmer E 1995 Intragenomic complementation of a 3AB mutant in dicistronic polioviruses. Virology 209:315–326. doi:10.1006/viro.1995.1263. 7778266

[38] Friebe P, Boudet J, Simorre JP, Bartenschlager R 2005 Kissing-loop interaction in the 3′ end of the hepatitis C virus genome essential for RNA replication. J Virol 79:380–392. doi:10.1128/JVI.79.1.380-392.2005. 15596831 PMC538730

[39] Zhang J, Yamada O, Yoshida H, Sakamoto T, Araki H, Shimotohno K 2007 Helper virus-independent trans-replication of hepatitis C virus-derived minigenome. Biochem Biophys Res Commun 352:170–176. doi:10.1016/j.bbrc.2006.10.188. 17112469 PMC7117360

[40] Frick DN 2007 The hepatitis C virus NS3 protein: a model RNA helicase and potential drug target. Curr Issues Mol Biol 9:1–20. 17263143 PMC3571657

[41] Gu M, Rice CM 2010 Three conformational snapshots of the hepatitis C virus NS3 helicase reveal a ratchet translocation mechanism. Proc Natl Acad Sci U S A 107:521–528. doi:10.1073/pnas.0913380107. 20080715 PMC2818896

[42] Crowder S, Kirkegaard K 2005 Trans-dominant inhibition of RNA viral replication can slow growth of drug-resistant viruses. Nat Genet 37:701–709. doi:10.1038/ng1583. 15965477

[43] Grassmann CW, Isken O, Tautz N, Behrens SE 2001 Genetic analysis of the pestivirus nonstructural coding region: defects in the NS5A unit can be complemented in trans. J Virol 75:7791–7802. doi:10.1128/JVI.75.17.7791-7802.2001. 11483722 PMC115021

[44] Khromykh AA, Sedlak PL, Westaway EG 2000 cis- and trans-acting elements in flavivirus RNA replication. J Virol 74:3253–3263. doi:10.1128/JVI.74.7.3253-3263.2000. 10708442 PMC111826

