# Supplementary material for: Polyprotein-Driven Formation of Two Interdependent Sets of Complexes Supporting Hepatitis C Virus Genome Replication
Source: J Virol. 2016 Feb 26;90(6):2868–83. doi: 10.1128/JVI.01931-15 (PMC4810661; doi:10.1128/JVI.01931-15)
Supplement: Supplemental material [file supp_90_6_2868__index.html]

Polyprotein-Driven Formation of Two Interdependent Sets of Complexes Supporting Hepatitis C Virus Genome Replication — Supplemental material 

# Polyprotein-Driven Formation of Two Interdependent Sets of Complexes Supporting Hepatitis C Virus Genome Replication

## Supplemental material

- Supplemental file 1 -

  Table S1 (Primer sequences.)

  PDF, 19K
