# Supplementary material for: Polyprotein-Driven Formation of Two Interdependent Sets of Complexes Supporting Hepatitis C Virus Genome Replication
Source: J Virol. 2016 Feb 26;90(6):2868–83. doi: 10.1128/JVI.01931-15 (PMC4810661; doi:10.1128/JVI.01931-15)
Supplement: Supplemental material [file JVI.01931-15_zjv999091410so1.pdf]

Table S1 Sequence of primers used in the study

Primer number	Primer sequence (5'-3')
1	CAGCTACAGCTATGTGACAG
2	GGTGGAGTCGAGGCCCAGC
3	CTAACCCACTGCTGGGCC
4	TGCAAGCGGCCAGGGCCTTC
5	TGCTGACAACCAGCATGGG
6	GTAAAACGACGGCCAG
7	GCTTGCTGGCAATCCACC
8	GCGCGCGCGTTTAAACTAGCACTCTTCCATTTCGTCG
9	GAGAGACGGTCCGAGCGGCTCTTGGCTGAGAGATG
10	CCAACCCCGGTCCGAGCATGAGCTACAGCTGGACA
11	CAGCACGGGAGGGGCTGTGCCCCAGACCTATC
12	GCACAGCCCCTCCCGTGCTGTTGTCACTGAAAG
13	GCAGTGGAAACAGCACCAAGGTCCCTGTCG
14	CTTGGTGCTGTTTCCACTGCCAGTTGGAGC
15	GCACGTGGGGCCTAGCTGGAGGAGTCCTGG
16	CCAGCTAGGCCCCACGTGCTGGTCATGACC
17	GAGGTCCTGGCTGAGGCTTTTGATGAGATG
18	CAAAAGCCTCAGCCAGGACCTCCTTATCCGGCG
19	GGATAACTGAGGACTGCCCCTCCCATGCCCCGGATCCTGG
20	ACACGTCGCGGAGCCAGGATCGGGGCATGGGATGGGGCAG
21	CCCCAAGCTGGCCGGCCTCCCCTTCATCTC
22	GGGAGGCCGGCCAGCTTGGGGAACAATTTAG
23	GTATGCGGCGCTGCCCTAGTAGTCATCTCAGAAAG
24	CTACTAGGGCAGCGCCGCATACCAGCATTG
25	CACGTACTCCGCATATGGCAAATTTCTCGC
26	ATTTGCCATATGCGGAGTACGTGATGGCCTCC
27	CACCGTTGCTGCTTGTGCTCTCCCACCTCCC
28	GAGAGCACAAGCAGCAACGGTGGGCGGTTGG
29	GCAGCGGCAACTCCACAAAAGTGCCTGTGG
30	CTTTTGTGGAGTTGCCGCTGCCGGTTGGGG
31	CAATCCCCTGCCCCGGCTCTTGGCTGAGAGATG
32	CAAGAGCCGGGGCAGGGGATTGGGCAATCCTC
33	GTGCGGCGCCGCTCTGGTCGTGATCAGCGAG
34	CGACCAGAGCGGCGCCGCACACGAGCATGG
35	CACCTACTCTGCCTACGGCAAGTTCCTGG
36	CTTGCCGTAGGCAGAGTAGGTGATAGCCTCG
37	GAGACATGTGGCCCCTGGCGAAGGCGCTGTGC
38	CTTCGCCAGGGGCCACATGTCTCCGCAGGATG
39	CAAGCACATGGGGGCTGGCTGGCGGAGTGCTG
40	CCAGCCAGCCCCCATGTGCTTGTCATCACTTC
41	CCAACCCGGGTCCGGCTCCCATCACTGCTTATGCCCAG
42	GAGGCGCTCTTTGATGTTGT
43	CAGCTACAGCTATGTGACAG
44	GTATGACATGGAACAACAAACTGTGGTATCATCGG
45	CAGTTTGTTGTTCCATGTCATACTCCTGGAC
46	CAGTTAGCTATGGAGTGTACC
